# Distance to parks and non-residential destinations influences physical activity of older people, but crime doesn’t: a cross-sectional study in a southern European city

**DOI:** 10.1186/s12889-015-1879-y

**Published:** 2015-06-27

**Authors:** Ana Isabel Ribeiro, Andrea Pires, Marilia Sá Carvalho, Maria Fátima Pina

**Affiliations:** Departamento de Epidemiologia Clínica, Medicina Preditiva e Saúde Pública, Faculdade de Medicina, Universidade do Porto, Rua do Campo Alegre, 823, 4150-180 Porto, Portugal; ISPUP – Instituto de Saúde Pública da Universidade do Porto, Rua das Taipas, 135, 4050-600 Porto, Portugal; i3S - Instituto de Investigação e Inovação em Saúde, Universidade do Porto, Porto, Portugal; INEB – Instituto de Engenharia Biomédica, Universidade do Porto, Rua do Campo Alegre, 823, 4150-180 Porto, Portugal; UFAL - Universidade Federal de Alagoas, Alagoas, Brazil; PROCC – Programa de Computação Científica, Fundação Oswaldo Cruz, Av. Brasil, 4365 - Antiga Residência Oficial, Manguinhos, 21045-900 Rio de Janeiro, RJ Brasil; ICICT/FIOCRUZ - Instituto de Comunicação e Informação Científica e Tecnológica em Saúde/Fundação Oswaldo Cruz, Rio de Janeiro, Brazil; CARTO-FEN/UERJ - Departamento de Engenharia Cartográfica, Faculdade de Engenharia da Universidade do Estado do Rio de Janeiro, Rio de Janeiro, Brazil

**Keywords:** Physical activity, Safety, Older adults, Parks, Destinations

## Abstract

**Background:**

Physical activity (PA) has numerous health benefits, but older adults live mostly sedentary lifestyles. The physical and social neighborhood environment may encourage/dissuade PA. In particular, neighborhood crime may lead to feeling unsafe and affect older adults’ willingness to be physically active. Yet, research on this topic is still inconclusive. Older population, probably the age group most influenced by the neighborhood environment, has been understudied, especially in Southern Europe. In this study, we aimed to analyze the association between leisure-time physical activity (LTPA) in older adults and objective crime, alongside other neighborhood characteristics.

**Methods:**

We obtained data from a population-based cohort from Porto (2005–2008) to assess LTPA. Only adults aged 65 years or more were included (*n* = 532). A Geographic Information System was used to measure neighborhood characteristics. Neighborhood crime was expressed as crime rates by category (incivilities, criminal offenses with and without violence and traffic crime). Neighborhood characteristics such as socioeconomic deprivation, land gradient, street density, transportation network, distance to parks, non-residential destinations and sport spaces were also included. Generalized Additive Models were fitted to estimate the association between neighborhood characteristics and the participation (being active vs. inactive) and frequency (min/day) of LTPA.

**Results:**

Forty-six percent of the men and 61 % of the women did not engage in any kind of LTPA. Among the active participants, men spent on average 50.5 (35.2 Standard Deviation, SD) min/day in LTPA, whereas the average among women was 36.9 (35.1 SD) min/day (*p* < 0.001).

Neighborhood crime was unrelated to the participation in, or frequency of, LTPA. On the other hand, two neighborhood characteristics – distance to the nearest park (β = −0.0262, *p* = 0.029) and to the nearest non-residential destination (β = −0.0735, *p* = 0.019) – were associated with time spent on LTPA, but only among active older women. No neighborhood characteristic was related to participation in LTPA.

**Conclusions:**

From a public health point of view, the provision of parks and non-residential destinations (shops, schools, cultural and worship places) might contribute to elevate PA levels of already active older women. On the other hand, in this setting, crime was not a big issue.

**Electronic supplementary material:**

The online version of this article (doi:10.1186/s12889-015-1879-y) contains supplementary material, which is available to authorized users.

## Background

Physical activity (PA) has numerous health benefits [1], but most people, and especially older adults, lead sedentary lifestyles [2]. Due to the increasing share of older populations in our societies [3], understanding the correlates of PA in this demographic group has never been so important. Physical activity habits are influenced by a myriad of aspects, including the social and physical environment [4]. The last two decades have been fertile in studies trying to determine the association between physical and social characteristics of the neighborhood and PA among older adults. But research on this topic is still not conclusive [5-7]. Literature shows mixed associations between different aspects of the neighborhood environment (access to parks/sport spaces or destinations, deprivation, land-uses, aesthetics) and PA [5-7].

Crime is one neighborhood characteristic that can act as a barrier to physical activity [8]. It is likely that people living in neighborhoods with high crime rates feel unsafe and, consequently, they might avoid engaging in PA in the neighborhood. Despite being a scientifically sound theory, neighborhood crime is one of the environmental correlates of PA that has led to more inconsistent and counterintuitive findings [9]. Perceived (self-reported) and objective (police recorded) measures of crime have been used in studies about this issue. The two provide distinct and complementary information [10], while objective crime expresses the likelihood of a crime occurring, perceived crime captures the individual interpretation of this tangible reality. Ideally, both perceived and objective crime should be addressed. Yet, studies using objective measures are particularly helpful because they are based on concrete indicators, making it easier to translate research findings into interventions that promote active lifestyles [11].

Older people have been subject to a limited number of studies relating crime and PA. In 2008, Foster and Giles-Corti reviewed all evidence about the topic and found that only 6 out of 41 studies have focused on samples of older adults [9]. Older adults are particularly vulnerable to the effects of neighborhood environments [12] and, principally older women, are more fearful of crime than any other demographic group [9,13-15]. Moreover, these studies have mostly used perceived measures of neighborhood crime [16-20] and as for adult samples, the results are not consistent – some detect significant associations [18-21] but others do not [16,17]. Further studies have since been published but the evidence remains limited: mixed results (6 studies detected some kind of association [10,22-26], but in 3 no association at all [27-30]); objective measures of crime were lacking [10,22,23,27]; and not all have dissected the effects of different categories of crime [10,23] (which might obscure the specific effect of some crime types).

Regardless of the neighborhood characteristics under analysis, Southern Europe has been neglected. Populations in Southern European countries rank among the oldest and most inactive in Europe [31,32]. Portugal, specifically, has one of the highest proportion of respondents saying they never exercise or play sport – 64 % of the adults (≥18 years) [31]. Populations residing in these areas therefore need further attention.

To address these gaps, we aimed to study the association between leisure-time physical activity (LTPA) among older adults and objective crime, without disregarding other neighborhood characteristics. Data will be drawn from a population-based cohort of adults residing in Porto (Portugal), and a wide range of objectively measured neighborhood characteristics will be used.

## Methods

### Setting

Located in the northwest of Continental Portugal, Porto municipality had approximately 240,000 inhabitants in 2008 [33], distributed across 41.7 km^2^. Porto is limited by the Atlantic coast, and extends along the Douro River estuary. It is an industrial and port town situated in the Porto Metropolitan Area, the second largest metro area of Portugal with roughly 1.3 million inhabitants [34].

### Participants

The EPIPorto Cohort encompasses a representative sample of 2485 adult (≥18 years old) inhabitants of Porto. Baseline evaluation was conducted from 1999–2003 [35]. Participants were recruited by random digit dialing using households as the sampling unit. After assessing the number and age of the residents of each household, randomization was applied to select one eligible person among the permanent adult residents.

The follow-up evaluation took place from 2005–2008. 1943 participants were contacted but 261 participants refused to participate, resulting in a response rate of 86.6 %.

The Ethics Committee of the Hospital de São João approved the study protocol. The study was carried out according to the Helsinki Declaration and all participants completed the informed written consent form.

Google Earth™ was used to georeference all addresses. For the present study, we included only adults aged 65 or more at the follow-up evaluation, i.e., 582 out of 1682 participants. Five participants were excluded because they moved outside of Porto.

### Outcome: Leisure-time physical activity

Physical activity was evaluated using the EPIPorto Physical Activity Questionnaire to measure time and intensity of different types of activities, such as rest, transport to/from work, occupational, household and leisure [36]. A previous study assessed the validity, reproducibility and seasonal bias associated with past-year PA reporting, and it showed it is a valid and reproducible instrument for the brief assessment of different types of PA among adults.

In our study we focused on leisure time physical activities. In the EPIPorto Physical Activity Questionnaire, these included sedentary (playing cards, watching TV), light (e.g. brisk walking, golfing, snooker), moderate (e.g. walk at moderate pace, dancing, stretching) and vigorous (e.g. running, soccer, basketball) leisure activities. Because older adults benefit from PA even if light [37], we considered LTPA as the sum of the time (minutes/day) spent in non-sedentary leisure activities.

Two measures of LTPA were defined: time spent (minutes/day) in LTPA and participation in LTPA – inactive (0 min/day) and active (>0 min/day). We followed this approach because we theorized that the time active individuals spend in LTPA might be more influenced by neighborhood characteristics, whereas participation in LTPA might be more related to individual characteristics than to the neighborhood’s [38].

Information about LTPA was available for 533 participants (out of 577), but one outlier observation had to be excluded, making a final sample of 532 participants.

### Covariates: Individual variables

Individual characteristics were obtained through a structured questionnaire. We considered as confounders the following individual correlates of LTPA: age; marital status (married/non-marital union, single, widowed and separated/divorced); educational attainment (number of schooling years); retirement status (not retired/retired); smoking status (smoker, occasional smoker, non-smoker and ex-smoker); comorbidities (absence/presence of at least one of the following conditions – cardiovascular, respiratory, osteoarticular and musculoskeletal disorders, cancer, depression, cirrhosis and hypo/hyperthyroidism); residence in Porto for 20 years or more (yes/no); and body mass index (classified according to World Health Organization cut-offs).

### Covariates: environmental variables

Neighborhood characteristics included as independent variables in the statistical analysis were: 1) socioeconomic status (SES) of the census tract of residence (three classes from the most to the least deprived [39]); 2) population density of the census tract of residence; 3) distance from the residence to the nearest park (24 parks); 4) distance to the nearest sport space (71 sport spaces); 5) distance to the nearest non-residential destination (includes churches, shops, libraries, museums and other points of interest) (421 non-residential destinations); 6) distance to the sea/riverside; 7) density of street intersections within 200 m of the residence (considered as the walkable distance for older individuals); 8) density of bus/metropolitan stops within 200 m; 9) average land gradient within 200 m. Since individual data refer to follow-up evaluation (2005–2008), all neighborhood characteristics were collected for a year within this time-window. The collection of the above mentioned variables and the georeferencing procedures were previously described [38].

**Fig. 1:**
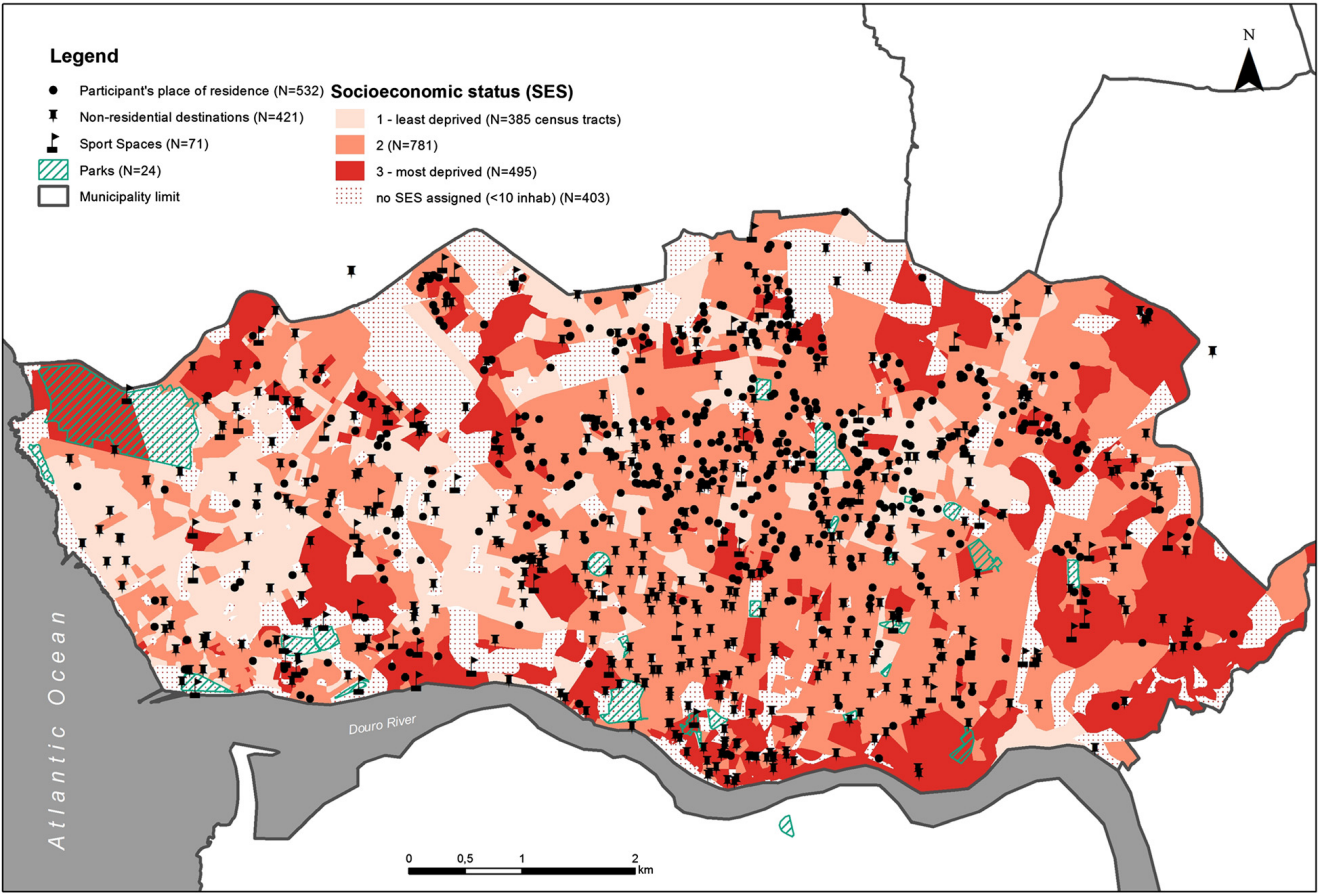
Spatial distribution of the participants’ residences and built and socio-environmental features (Porto, 2005–2008)

The map of the participants’ residence and neighborhood characteristics is displayed in Fig. 1.

### Covariates: crime

Data about crime were obtained from the Public Security Police of the Metropolitan Command of Porto, which provided records of all crimes in Porto during 2008. The dataset included a description of the crime and the place of occurrence (street, neighborhood, street segment and, occasionally, exact position).

There were 17,790 records, from which 296 could not be georeferenced due to poor quality location information and 1776 were excluded because they corresponded to crimes (e.g. fraud, jobbery, copyright crimes) that were unlikely have an impact on the population’s fear of crime and, consequently, PA.

Based on previous studies [10,23], we classified the remaining 15,718 crimes into the following categories: 1) incivilities (drug, vandalism, prostitution); 2) criminal offenses with violence, i.e., with approach to the victim (robbery, homicide, rape); 3) criminal offenses without violence, i.e., with no approach to the victim (theft, verbal offences) and 4) traffic (drunk/dangerous driving, speeding).

Further details about the georeferencing procedures and categorization of crime records can be found as additional material (additional file 1 and 2).

**Fig. 2:**
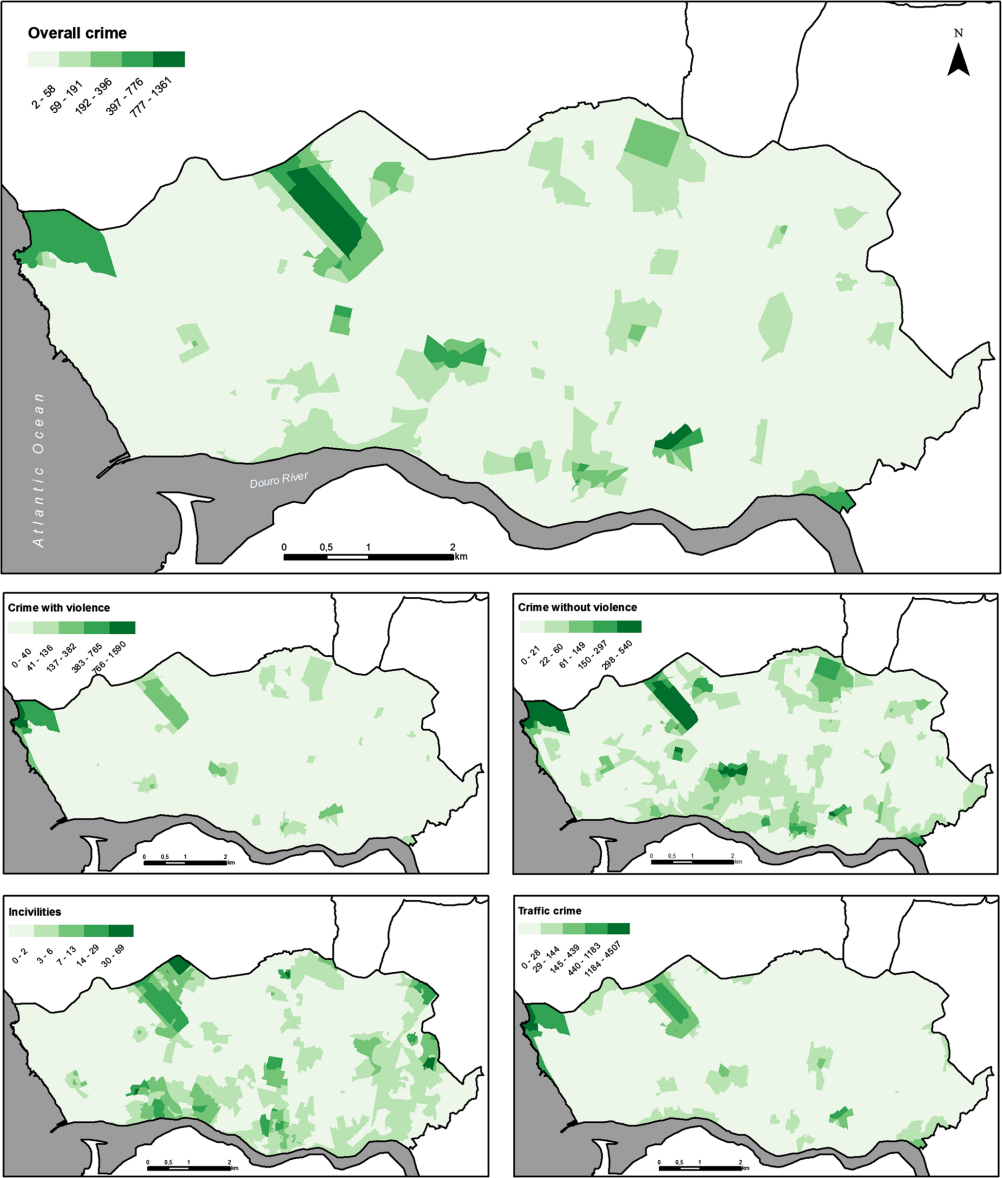
Spatial distribution of recorded crime (Porto, 2008). *Spatial distribution of the crime rates (crimes/1000 inhabitants) by category*

We calculated crime rates (/1000 inhabitants), by category, for each census tract; then a crime rate was attributed to each participant. Fig. 2 shows the spatial distribution of crimes rates across Porto municipality by category.

### Statistical analysis

Descriptive statistics were computed for all variables, by sex and participation in LTPA (active vs. inactive). Mann–Whitney U and Chi-square tests were employed to compare distributions and proportions; the significance level was set at 0.05.

Generalized Additive Models (GAM) were used to estimate the association between LTPA and covariates. GAM extends generalized linear models to include nonparametric smoothing. This approach allowed us to model the spatial distribution of LTPA, and therefore to control for the presence of possible spatial autocorrelation.

For data modeling, LTPA was used as a dependent variable and individual and neighborhood characteristics as covariates. Firstly, the association between spatial location of residence and LTPA was evaluated by applying a bivariate smoothing spline function on the pair of coordinates. Secondly, univariable analysis was conducted and all covariates with *p*-values ≤0.10 were included in the initial multivariable model. Then, each covariate was removed step by step until the final adjusted model was attained, eliminating consecutively those with the highest *p*-values. The final model included only covariates with *p*-values ≤0.05.

The presence of interactions was evaluated by including interaction terms between: 1) sex/marital status and area variables and 2) crime and other environmental variables.

Two models were fitted to test the hypotheses that 1) neighborhood characteristics were related to participation in LTPA and 2) neighborhood characteristics affect the time spent on LTPA among already-active persons. The first model, logistic regression, (eq 1) included the whole sample and assessed LTPA as a dichotomous variable (active/inactive). The second, linear regression, (eq 2) contained only active individuals, and assessed LTPA as a continuous variable (minutes/day). Given its skewed distribution, the variable LTPA (minutes/day) was log-transformed. The equations are presented below:

1 $$ logit\left({y}_i\right)={\beta}_0+{\displaystyle \sum }{\beta}_k{x}_{ik}+f\left( nort{h}_i,eas{t}_i\right)+{e}_i $$

2 $$ {z}_i={\beta}_0+{\displaystyle \sum }{\beta}_k{x}_{ik}+f\left( nort{h}_i,eas{t}_i\right)+{e}_i $$

where *y*_*i*_ and *z*_*i*_ are the response variables, *β* ' *s* are the coefficients of the model, *x*_*ik*_ are the explanatory variables, *f*(*north*_*i*_, *east*_*i*_) is a smooth function of the coordinates and *e*_*i*_ are the residuals.

Due to the presence of interactions between sex and some neighborhood characteristics, sex-stratified models were built.

## Results

### Sample characteristics

The characteristics of the participants are shown in Tables 1 and 2. The sample consisted of 39 % men, and the mean age was 72.7 (5.6 SD, standard deviation) and 73.7 (5.9 SD) years old, among men and women, respectively.

**Table 1:** Characteristics of the participants (Porto, 2005–2008) according to participation in LTPA (inactive or active)

	Total (*n* = 532)		Inactive (*n* = 294)		Active (*n* = 238)	
	Women (*n* = 323)	Men (*n* = 209)	Women (*n* = 198)	Men (*n* = 96)	Women (*n* = 125)	Men (*n* = 113)
	Mean (SD^a^) or No. (%)	Mean (SD) or No. (%)	Mean (SD) or No. (%)	Mean (SD) or No. (%)	Mean (SD) or No. (%)	Mean (SD) or No. (%)
Age (yrs)	72.7 (5.6)	73.7 (5.8)	73.3 (6.0)	74.0 (5.7)	71.8 (4.7)	73.4 (6.0)
Marital Status*:						
Married/un-married union	142 (44.0)	182 (87.1)	92 (46.5)	82 (85.4)	50 (40.0)	100 (88.5)
Single	24 (7.4)	1 (0.5)	15 (7.6)	1 (1.0)	9 (7.2)	0 (0.0)
Widowed	140 (43.3)	23 (11.0)	82 (41.4)	12 (12.5)	58 (46.4)	11 (9.7)
Divorced/separated	17 (5.3)	3 (1.4)	9 (4.5)	1 (1.0)	8 (6.4)	2 (1.8)
Education attainment (no. years)***	5.5 (4.1)	7.3 (4.4)	4.8 (3.7)	6.6 (4.0)	6.6 (4.4)	7.9 (4.5)
Retirement status*:						
Not retired	62 (19.2)	8 (3.8)	39 (19.7)	4 (4.2)	23 (18.4)	4 (3.5)
Retired	261 (80.8)	201 (96.2)	159 (80.3)	92 (95.8)	102 (81.6)	109 (96.5)
Residence in Porto (<20 years)	7 (2.2)	4 (1.9)	6 (3.0)	2 (2.1)	1 (0.8)	2 (1.8)
Comorbidities*:						
No	73 (22.7)	69 (33.0)	45 (22.8)	36 (37.5)	28 (22.4)	33 (29.2)
Yes	249 (77.3)	140 (67.0)	152 (77.2)	60 (62.5)	97 (77.6)	80 (70.8)
Body Mass Index***:						
Underweight (<18.5)	0 (0.0)	0 (0.0)	0 (0.0)	0 (0.0)	0 (0.0)	0 (0.0)
Normal (18.5-24.9)	69 (21.6)	70 (34.1)	39 (20.0)	23 (25.0)	30 (24.0)	47 (41.6)
Overweight (25.0-29.9)	136 (42.5)	102 (49.8)	77 (39.5)	51 (55.4)	59 (47.2)	51 (45.1)
Obese (≥30.0)	115 (35.9)	33 (16.1)	79 (40.5)	18 (19.6)	36 (28.8)	15 (13.3)
Smoking habits***:						
Smoker	6 (1.9)	19 (9.1)	5 (2.6)	5 (5.2)	1 (0.8)	14 (12.4)
Occasional smoker	1 (0.3)	2 (1.0)	1 (0.5)	1 (1.0)	0 (0.0)	1 (0.9)
Non-smoker	290 (90.3)	72 (34.4)	182 (92.9)	31 (32.3)	108 (86.4)	41 (36.3)
Ex-smoker	24 (7.5)	116 (55.5)	8 (4.1)	59 (61.5)	16 (12.8)	57 (50.4)
LTPA^b^ (minutes/day)***	14.3	27.3	0.0 (0.0)	0.0 (0.0)	36.9 (35.1)	50.5 (35.2)
	(28.2)	(36.1)				

*p ≤ 0.05 comparing men and women

**p ≤ 0.05 comparing active and inactive

^a^ *SD* standard deviation

^b^ *LTPA* leisure-time physical activity

**Table 2:** Characteristics of the participants’ neighborhood environment (Porto, 2005–2008) according to participation in LTPA (inactive or active)

	Total (*n* = 532)		Inactive (*n* = 294)		Active (*n* = 238)	
	Women (*n* = 323)	Men (*n* = 209)	Women (*n* = 198)	Men (*n* = 96)	Women (*n* = 125)	Men (*n* = 113)
	Mean (SD^a^) or No. (%)	Mean (SD) or No. (%)	Mean (SD) or No. (%)	Mean (SD) or No. (%)	Mean (SD) or No. (%)	Mean (SD) or No. (%)
Distance to the nearest parks (hm)	9.9 (6.4)	10.9 (6.6)	9.7 (6.2)	10.9 (7.1)	10.3 (6.6)	10.8 (6.2)
Distance to the nearest sport space (hm)	10.0 (4.7)	6.6 (3.5)	9.7 (4.7)	6.6 (3.4)	10.4 (4.7)	6.7 (3.5)
Distance to the nearest non-residential destination (hm)	3.3 (2.2)	3.5 (2.3)	3.3 (2.1)	3.4 (2.3)	3.3 (2.5)	3.5 (2.3)
Distance to the sea/riverside (hm)	33.9 (11.0)	32.6 (11.5)	34.7 (11.4)	33.0 (11.4)	32.7 (10.5)	32.3 (11.7)
Intersection density^b^ (nodes/ha)	12.3 (6.7)	12.5 (6.8)	12.7 (6.9)	12.2 (6.4)	11.6 (6.3)	12.7 (7.2)
Bus/metropolitan stops (no.)^b^	3.4 (1.9)	3.2 (1.9)	3.5 (1.9)	3.3 (2.1)	3.2 (1.8)	3.2 (1.7)
Land gradient (%)^b^#	5.0 (3.6)	4.8 (3.2)	5.1 (3.5)	4.9 (3.1)	4.7 (3.7)	4.8 (3.3)
Population density (inhab./km2)^b^	13549.1 (9208.9)	13270.3 (9071.5)	13795.7 (9869.8)	13976.6 (10415.9)	13158.6 (8075.0)	12670.3 (7746.7)
Neighborhood SES^c^*:						
1 – least deprived	66 (20.4)	48 (23.0)	37 (18.7)	16 (16.7)	29 (23.2)	32 (28.3)
2 – medium deprived	202 (62.5)	123 (58.9)	122 (61.6)	58 (60.4)	80 (64.0)	65 (57.5)
3 – most deprived	55 (17.0)	38 (18.2)	39 (19.7)	22 (22.9)	16 (12.8)	16 (14.2)
Neighborhood crime (crimes/1000 inhab.):						
Incivilities	0.4 (0.8)	0.4 (0.5)	0.5 (1.0)	0.4 (0.4)	0.4 (0.5)	0.4b(0.6)
Crime without violence	22.4 (20.4)	20.9 (21.2)	20.3 (16.6)	21.8 (23.3)	25.7 (25.0)	20.1 (19.4)
Crime with violence	5.9 (7.5)	6.0 (8.3)	6.7 (8.6)	6.1 (10.0)	4.7 (5.1)	5.8 (6.5)
Traffic crime	7.5 (17.2)	7.1 (13.2)	7.7 (19.1)	6.2 (10.7)	7.3 (13.7)	7.8 (14.9)
Overall crime	26.9 (34.0)	25.9 (26.7)	29.6 (40.3)	24.7 (26.6)	22.7 (19.7)	26.9 (26.9)

*p ≤ 0.05 comparing active and inactive

^a^ *SD* standard deviation

^b^Within 200 m circular buffer

^c^ *SES* neighborhood socioeconomic status

Forty-six percent of the men and 61 % of the women do not engage any kind of LTPA. Among the active participants, men spend on average 50.5 (35.2 SD) min/day in LTPA, whereas women’s average is 36.9 (35.1 SD) min/day (*p* < 0.001).

Men and women differ significantly in several aspects. Compared with women, among men we observed higher educational attainment, a lower proportion of chronically ill, obese and widowed, and a higher proportion of smokers.

Active participants were more educated and less likely to be obese than inactive individuals.

Regarding the neighborhood characteristics, on average, participants had parks, sport spaces and non-residential destinations within a distance shorter than 1000 m from their residence. The average street intersection density was 12 nodes/ha, and participants had on average 3 bus stops in a radius of 200 m around their residence. Most of the participants (61 %) were classified as medium SES neighborhoods.

The majority of the crimes (57 %) corresponded to criminal offenses without violence (*circa* 22 occurrences/1000 inhabitants) and the reporting of incivilities was rare (*circa* 0.4/1000). After non-violent crime, traffic crime was the most common crime category (*circa* 7/1000), followed by criminal offenses with violence (*circa* 6/1000).

Active and inactive participants did not differ in most neighborhood characteristics, except in relation to socioeconomic deprivation and land gradient, which seemed lower among active participants. Men and women did not differ in any of the neighborhood characteristics.

### Role of neighborhood environment on LTPA

We observed no spatial autocorrelation in the distribution of LTPA (either active/inactive or min/day). Consequently, the spatial smoothing term was excluded from the models.

When considering the whole sample and the response variable as participation in LTPA (active vs. inactive), logistic regression models revealed no association between crime (and any other neighborhood characteristics) and participation in LTPA among men. We only found a significant association between participation in LTPA and the rates of non-violent crime (Odds Ratio, OR = 1.019; IC95% = 1.004–1.027, *p* = 0.014) among women.

Concerning the outcome as time spent in LTPA by active individuals, the results (Table 3) show the adjusted and unadjusted coefficients for the association between neighborhood characteristics and time spent by active individuals in LTPA. There was no significant association between crime and time spent in LTPA, regardless of the category. We also tested for interactions and found no significant association.

**Table 3:** Association between time spent in leisure-time physical activity of active participants and neighborhood characteristics. Association between daily minutes spent in leisure-time physical activity (log-transformed) of active participants and neighborhood characteristics, stratified by sex (Porto, 2005–2008)

	Model 1^a^				Model 2^b^			
	Women		Men		Women		Men	
	Coefficient	*p-value*	Coefficient	*p-value*	Coefficient	*p-value*	Coefficient	*p-value*
Distance to the nearest park (hm)	−0.0275	0.017	−0.0063	0.573	−0.0262	0.029		
Distance to the nearest sport space (hm)	−0.0297	0.068	0.0471	0.017			0.0462	0.032
Distance to the nearest non-residential destination (hm)	−0.0750	0.014	0.0125	0.680	−0.0735	0.019		
Distance to the sea/riverside (hm)	−0.0031	0.669	−0.0011	0.852				
Intersection density^c^ (nodes/ha)	−0.0073	0.549	−0.0070	0.471				
Bus/metropolitan stops (no.)^c^	0.0093	0.828	0.0089	0.823				
Land gradient (%)^c^	−0.0254	0.221	−0.0102	0.628				
Population density (inhab./ha)^c^	0.0006	0.495	−0.0005	0.596				
Neighborhood SES^d^								
1 – least deprived	Ref		Ref					
2 – medium deprived	−0.0394	0.832	0.0242	0.879				
3 – most deprived	−0.1358	0.612	0.1921	0.393				
Neighborhood crime (crimes/1000 inhab.)								
Incivilities	−0.0008	0.996	−0.0008	0.995				
Crime without violence	−0.0015	0.615	0.0029	0.423				
Crime with violence	−0.0081	0.593	0.0012	0.991				
Traffic crime	0.0045	0.422	0.0020	0.669				
Overall crime	−0.0156	0.689	0.00038	0.883				

^a^Univariable regression

^b^Multivariable regression adjusted for age, educational attainment, marital status, retirement status, residence in Porto for 20 years or more, comorbidities, BMI and smoking habits

^c^Within 200 m circular buffer

^d^ *SES* neighborhood socioeconomic status

However, significant associations with other neighborhood characteristics were observed. In the univariable analysis, among women, distances to the nearest park and to non-residential destination were negatively associated with the time spent in LTPA. After adjustment, associations between the distance to the nearest park (β = −0.0262, *p* = 0.029) and non-residential destination (β = −0.0735, *p* = 0.019) remained. That is, for every 100 m increase in the distance to the nearest park and non-residential destination, the time spent in LTPA reduces ((1 − *e*^*β*^) × 100) by 2.6 % and 7.1 %, respectively.

In men, we observed a positive association between distance to nearest sport space and LTPA (β = 0.0462, *p* = 0.032).

The proportion of the explained variability in LTPA (minutes/day) of the linear models was 17.1 % for women and 10.9 % for men; higher than in the logistic model (active/inactive), where it did not surpass 10 % for women and 7 % for men.

## Discussion

Our study represents one of the most comprehensive studies of neighborhood influences on physical activity among older adults from southern Europe, and the first addressing the impact of neighborhood crime. We found neighborhood crime was unrelated to the practice or the frequency of LTPA. On the other hand, we observed that other neighborhood characteristics – distance to the nearest park and to the nearest non-residential destination – were associated with the time spent on LTPA, but only among older women that were active in some way. These characteristics were also unrelated to whether they were physically active or not.

Regarding the role of our primary neighborhood variable, objective crime, results did not corroborate our hypothesis. No main or interaction effects between neighborhood crime (and its categories) and PA were found. We only found a positive association between participation in LTPA and non-violent crimes among women.

Several studies have reported that crime, dissuades seniors from being active [10,18-23,25,26,40]. The fewer studies using objective measures of crime [10,21-23] actually provide evidence for such an association, whereas within the group of studies based on measures of perceived crime [16-20,25-30,40], null associations were frequent [16,17,27-30]. The fact we could not identify significant associations between PA and neighborhood crime might result from three possible explanations: (i) low risk of crime; (ii) walkable neighborhoods are attractive to crime; and (iii) social/cultural factors alleviate feeling unsafe.

Porto, like most Portuguese cities, is a relatively safe urban area and the few existing threats might not suffice to dissuade older adults from engaging PA. Portugal is at the bottom half in the rank of the European Crime Statistics, having lower crime rates than the UK, France or Spain [41]. The studies we found about the role of objective crime on older adults PA were undertaken in different countries and/or cities (USA, Oslo and Amsterdam), where crime might be a bigger issue.

Another plausible explanation lies with the fact that the same areas which provide destinations to walk do also provide opportunities for crime. The resources that define a walkable neighborhood – presence of shops, recreational facilities, dense transportation network, street connectivity, and food and alcohol outlets – have been associated with higher levels of crime [42-45]. Therefore, the negative influence that crime exerts on PA might be silenced by the positive impact of living in a walkable neighborhood. A recent study demonstrated that this seems to be a very plausible explanation of the null or counterintuitive findings found in studies about the effects of neighborhood crime on PA [46]. Notice that we found a positive association between neighborhood crime and PA in women, which happens to be the same demographic group whose PA levels increased with the proximity to non-residential destinations (shopping centers, recreational places). In our study we sought evidence for interactions between crime and other characteristics but we were not able to detect any, not even between neighborhood crime and distance to non-residential destinations.

Finally, another possible reason of the null associations might derivate from the specificity of the Portuguese social context. Social interactions and strong family ties in Portugal, and other Southern European countries, tend to be more common than in northern countries (where most studies have been performed) [47-49]. Studies have shown that perceived safety and self-efficacy might be determined by social support within the family and community [50,51].

In our study we also found no evidence that neighborhood characteristics significantly influence whether older adults are physically active or not. That represents no novelty for us. In a previous study, using baseline data (1999–2003) from the same population-based cohort, we found that neighborhood characteristics did not define whether older adults were active (some PA) or inactive (no PA at all). As in the present study, access to parks and non-residential destinations was only relevant among the elderly who already participate in PA [38]. Very few studies have looked at LTPA this way (both as dichotomous and continuous variables) but two processes are involved here and should be analyzed separately: participation in any LTPA at all and the amount of time dedicated to LTPA. Physical activity (and other health-related behaviors) is chiefly shaped at early life-stages and depends upon personal characteristics (e.g., sociocultural and educational aspects, or even physician recommendation) [52,53]. Thus, it would be unlikely that neighborhood environments effect an older person who has never exercised in his/her entire life. On the contrary, for those that already exercise on a daily basis, having an extra exercise facility in their neighborhood might increase their levels.

On the other hand, the associations we found between LTPA and proximity to parks and non-residential destinations corroborate the literature on the topic. The role of parks in PA has been extensively studied and it seems that access to parks may encourage people to engage in PA by, for example, providing increased opportunities for walking and cycling [20,54-56]. Similarly, access to non-residential destinations (sometimes expressed as land-use mix) has been consistently associated with increased PA among the elderly [25,28,56-59].

In our study, these associations were exclusive to women. The explanatory capability of our models, although modest, was higher in women (17 %) than in men (11 %), implying neighborhood characteristics have lesser impact on men’s choices and attitudes toward PA. Accumulated knowledge on this topic suggests that residential environments might be more important for women's health and health-related behaviors than for men’s [60].

In men, we found a positive association between distance to the nearest sport space and time spent in LTPA – those living farther away spending more time. A possible explanation for that unexpected finding would be the presence of unaccounted characteristics near sport spaces that dissuade PA (such as noise, pollution, social capital). As previously stated, we believe that among men, individual motivation and social support (e.g. having friends around to play with) might be much more relevant in shaping their PA habits than neighborhood characteristics.

### Limitations

Our study has some limitations to consider. First, the cross-sectional nature of the study does not allow us to prove causal associations, due to the possibility of reverse causation and unmeasured confounding. Secondly, although we included a wide range of neighborhood characteristics, we could not incorporate characteristics known to affect PA, such as traffic [58], aesthetics [61] and social support [25,61]. Due to data unavailability, the role of perceived neighborhood environment, namely perceived crime, could not be explored. Third, we relied on self-reported PA, which might lead to recall and reporting bias. However, the EPIPorto PA Questionnaire was based on a well-established questionnaire and the validation procedure showed that it is a valid and reproducible instrument for assessing PA among adults [36]. Fourth, our measure of neighborhood crime might present some limitations as well. Objective crime refers to a single year (2008) and, although the overall crime rates did not change significantly in the proximate years, we cannot exclude the hypothesis that small space-time fluctuations occurred. In that circumstance, the use of crime records from other years/periods could have produced different results. Moreover, we cannot rule out the possibility that the crime records’ accuracy varied by neighborhood, which could lead to individuals’ differential misclassification.

### Strengths

Our study has several strengths too. It represents one of the most comprehensive studies of the neighborhood influences on physical activity among older adults from southern Europe, and the first addressing the impact of neighborhood crime. The effects of neighborhood environments on PA might be context- and culture-specific. Consistency is one of the key criteria for causation: consistent findings observed by different persons in different places with different samples strengthens the likelihood of an effect [62]. Moreover, as previously referred to, the lowest levels of physical activity are clustered in Southern Europe and current economic constraints can only contribute to exacerbate this position [31]. Studies like ours might lead to interventions in urban design, which will improve population PA levels without being too costly - an important aspect when economic resources are limited. Secondly, we used a vast range of objectively measured neighborhood characteristics, minimizing bias due to unaccounted confounding variables. Third, crime was divided into different categories allowing us to determine the impact of each. Finally, our study contributes to consolidate the knowledge on an important, and still unsolved, public health issue – what are the urban environment correlates of PA? We believe the answer to that real-world question will lead to significant changes in urban planning policies.

## Conclusions

We found no association between objective crime and the participation, and frequency of, LTPA among older adults. On the other hand, two neighborhood characteristics – distance to non-residential destinations and parks – were related to the time spent in LTPA, but only among older women that were active in some way. We also found no evidence that neighborhood characteristics define physical activity habits – being active (some PA) or inactive.

From a public health point of view, the provision of non-residential destinations such as shops, cultural and worship places, schools and parks might contribute to elevate PA levels of already active seniors. Yet, a profound change of PA habits might require multifaceted strategies that include environmental modifications, but also individual guidance provided by physicians, educators and mass media.

## Additional files

Additional file 1:
**Georeferencing of crime records.**
Additional file 2:
**Categorization of crime records.**
