# Supplementary material for: Distance to parks and non-residential destinations influences physical activity of older people, but crime doesn’t: a cross-sectional study in a southern European city
Source: BMC Public Health. 2015 Jun 27;15:593. doi: 10.1186/s12889-015-1879-y (PMC4483219; doi:10.1186/s12889-015-1879-y)
Supplement: Additional file 1: — Georeferencing of crime records. [file 12889_2015_1879_MOESM1_ESM.pdf]

Additional material 1: Georeferencing of crime records

Georeferencing of crime records took three steps:

1) Standardization of street names and correction of spelling errors;
2) Georeferencing of crime records either in the rooftop of the building (when the complete address was available), in the centroid of the street segment (when the door number interval of the street segment of occurrence was available) or in the centroid of the street (when the name of the street of occurrence was the only information available)
3) Counting of crime frequency (by category) in each location;

Because we needed a measure of crime for each census tract (i.e. neighborhood) the following procedures were required:

4) Creation of a buffer of 100 meters around each crime location and intersection with census tract (for which we had population data) to assess the population within each buffer.
5) Computation of crime rates in each buffer as following: $\frac{total\ crimes\ within\ the\ buffer}{population\ within\ the\ buffer} * 1000$
6) Because frequently more than one buffer intersected with each census tract, we choose to attribute to the census tract the highest crime rate of the buffers that intersect it.
