# Supplementary material for: Distance to parks and non-residential destinations influences physical activity of older people, but crime doesn’t: a cross-sectional study in a southern European city
Source: BMC Public Health. 2015 Jun 27;15:593. doi: 10.1186/s12889-015-1879-y (PMC4483219; doi:10.1186/s12889-015-1879-y)
Supplement: Additional file 2: — Categorization of crime records. [file 12889_2015_1879_MOESM2_ESM.pdf]

Additional material 2: Categorization of crime records

Category	Description	Crime	
Traffic (n=2,270)	Crimes associated with the violation of traffic rules and road safety.	• Driving under the influence (1211)<br>• Unlicensed driving (1015)<br>• Reckless or dangerous driving (36)	• Assault in traffic accident (7)<br>• Homicide in traffic accident (1)
Incivilities (n=492)	Mild forms of misbehavior as public drunkenness, delinquent behavior and situations that cause disorder and sense of public insecurity.	• Drug traffic (354)<br>• Possession or traffic of prohibited weapons (64)<br>• Other offenses related to drugs (60)	• Heritage damage (3)<br>• Drug possession (10)<br>• Child possession and prostitution (1)
Criminal offenses without violence (n=8,922)	Menacing crimes without victim approach	• Theft in motor vehicle (3,122)<br>• Theft in residence with burglary, scaling or false keys (1404)<br>• Theft by pickpocket (967)<br>• Theft of motor vehicle (913)<br>• Other thefts (774)<br>• Theft in commercial or industrial building with burglary, scaling or false keys (676)<br>• Threat or coercion (577)<br>• Theft in supermarket (173)	• Coercion-resistance of worker (120)<br>• Theft in other buildings, with burglary, scaling or false keys (89)<br>• Theft in the school, with burglary, scaling or false keys (63)<br>• Burglary (38)<br>• Theft and traffic of art and culture (3)<br>• Child abduction (2)<br>• Human traffic (1)
Criminal offenses with violence (n=4,034)	Menacing crimes with victim approach	• Simple assault (1200)<br>• Domestic violence against partner (1125)<br>• Robbery in the street (787)<br>• Other domestic violence offenses (375)<br>• Theft/pickpocketing (302)<br>• Other robbery (141)<br>• Aggravated assault (29)<br>• Domestic violence against minors (23)<br>• Robbery of public transport driver (13)	• Taking, confinement, or restrain (8)<br>• Intetional homicide (7)<br>• Child abuse (6)<br>• Rape (6)<br>• Child sexual abuse (5)<br>• Other assault offemses (4)<br>• Bank robbery (2)<br>• Service station robbery (1)
